# The Role of Academic Health Systems in Leading the “Third Wave” of Digital Health Innovation

**DOI:** 10.2196/32679

**Published:** 2022-11-09

**Authors:** Adeel A Faruki, Richard D Zane, Jennifer L Wiler

**Affiliations:** 1 Department of Anesthesiology University of Colorado Hospital School of Medicine Aurora, CO United States; 2 Department of Emergency Medicine University of Colorado Hospital School of Medicine Aurora, CO United States

**Keywords:** innovation, academic hospitals, academic health systems, health technology, entrepreneur, disruption, digital health, research programs, cost, investment, intrapreneur

## Abstract

Investors, entrepreneurs, health care pundits, and venture capital firms all agree that the health care sector is awaiting a digital revolution. Steven Case, in 2016, predicted a “third wave” of innovation that would leverage big data, artificial intelligence, and machine learning to transform medicine and finally achieve reduced costs, improved efficiency, and better patient outcomes. Academic medical centers (AMCs) have the infrastructure and resources needed by digital health intrapreneurs and entrepreneurs to innovate, iterate, and optimize technology solutions for the major pain points of modern medicine. With large unique patient data sets, strong research programs, and subject matter experts, AMCs have the ability to assess, optimize, and integrate new digital health tools with feedback at the point of care and research-based clinical validation. As AMCs begin to explore digital health solutions, they must decide between forming internal teams to develop these innovations or collaborating with external companies. Although each has its drawbacks and benefits, AMCs can both benefit from and drive forward the digital health innovations that will result from this journey. This viewpoint will provide an explanation as to why AMCs are ideal incubators for digital health solutions and describe what these organizations will need to be successful in leading this “third wave” of innovation.

## Introduction

In 2016, Steve Case, health care futurist, entrepreneur, investor, and former CEO of America Online, declared the health care industry ripe for disruption and predicted a “third wave” of innovation that would alter how we practice medicine [1]. This innovation would leverage big data, artificial intelligence, and machine learning to transform health care and finally achieve the reduced costs and improved outcomes demanded by the public. Case’s prediction appears imminent, but even extremely successful companies have already tried and failed to disrupt our complex health care system as seen with the joint venture Haven [2]. Ambitious ideas combined with a revenue of US $534 billion dollars and a high-profile leadership team could not tackle the perverse incentives and market dynamics of the current health care system [3]. Therefore, it is imperative that this change must develop within health care organizations.

Medical research insights and knowledge in health care overall have been rapidly expanding in the past 70 years due to advances in technology, and this momentum will only accelerate as early adoption of health care innovation continues [4]. As Balas and Chapman [5] stated, health care innovation is based on the criteria of “being novel, nonobvious and useful.” With this definition, innovation can be seen as a gradual process improvement by updating outdated processes and tools, or a complete disruption of current systems. Critically, innovation in health care will never achieve significant positive impact on patient outcomes without accepting the risks of early adoption and without the integration of digital health solutions into common practice. This viewpoint will further provide an explanation as to why academic medical centers (AMCs) are ideal incubators for digital health solutions; it will also describe what these organizations will need to be successful in leading this “third wave” of innovation.

## Why AMCs Should Lead the “Third Wave”?

AMCs across the United States serve as community hospitals, safety net institutions, and as state-of-the-art research-intensive quaternary referral centers. Even though AMCs only account for 6% of the US health system in 2012, they account for “47% of organ transplants, 60% of level one trauma centers, and 66% of burn units” [6,7]. These referral centers are known to provide care for the most complex patients with rare or difficult-to-manage disease processes and multiple comorbidities [8]. The combination of patients with disease processes at various stages and large catchment areas results in enormous amounts of unique data that are constantly growing and stored in relatively easily accessible electronic medical records. These large stores of unique patient data can be leveraged to ensure any digital health solutions developed are applicable to diverse patient populations. Historically, these data had primarily been abstracted by billing coders, nurses, cancer registrars, quality improvement teams, and health information management professionals at an institutional level [9]. The purposes of the abstraction have included billing, patient registry functions, quality improvement initiatives, and clinical research. We note that most health care institutions have the infrastructure to abstract data successfully from the electronic medical record (EMR) for digital health innovation; however, at AMCs, this can be performed with increased safeguards to safeguard patient’s protected health information through institutional review board (IRB)–approved research projects.

Research has always been one of the integral components of the mission of any academic institution and AMCs; medical schools and universities continue to be extensively funded to perform this mission with US $29.5 billion dollars of funding from the National Institutes of Health extramural awards in fiscal year 2019 alone [10-12]. Landmark medical developments from AMCs range from the HER2-Herceptin breast cancer treatment clinical trial at University of California Los Angeles in 2001 to the discovery of altered mRNA initiating a protective immune response at the University of Pennsylvania in 2005 [13-15]. These studies resulted in significant innovations in disease management or prevention and were the result of structured research projects at AMCs. The health care system, as a whole, has relied on AMCs to use basic science and clinical research, both funded and unfunded, to innovate in diagnostic modalities, therapeutics, care delivery, patient safety, and now in technology integration. AMCs have structured processes with technology transfer offices and IRBs to perform high-quality translational research. One significant benefit to any technology company who collaborates with an AMC will be the ability to clinically validate their digital health innovations through research studies that can be published as scientific manuscripts. This research supports the academic mission of the organization and acts as a differentiator for a health technology start-up company. Finally, AMCs have access to clinical faculty who deliver patient care on the frontline and can advise on those digital health implementations that will add the greatest value to clinical quality and patient safety. The collaboration between AMCs and technology companies has already started, but it has been a gradual process. Institutions such as the University of Pittsburgh Medical Center and University of California, San Francisco are already integrating artificial intelligence and machine learning into their EMR to help clinicians identify chronic diseases and improve imaging interpretation accuracy through co-development with external technology companies [16,17]. Moreover, UCHealth University of Colorado Hospital is performing clinical validation for a wireless, wearable patient motion sensor in the intensive care unit that communicates with the EMR to inform care teams if patients are at risk of pressure ulcer injuries [18]. Although, other types of health care organizations may have access to large stores of patient data and have legal infrastructure with contracting capabilities to collaborate with technology companies, AMCs have more extensive infrastructure to perform IRB-approved research studies. AMCs have experts in the field who are incentivized by their institutions to continually develop new knowledge, intellectual property, and novel technologies, which is why they are uniquely poised to lead the next wave of health care advancement through technology integration and validation.

## What Will AMCs Need to Lead the “Third Wave” of Digital Health Innovation?

To successfully lead in digital health innovation, AMCs will need to dedicate resources, both financial and human capital, to support these endeavors. The first determination is whether the AMC wants to develop digital health tools internally with their own employees acting as entrepreneurs within organizations, otherwise known as intrapreneurship, or through collaboration with external companies. Intrapreneurship, as explained by Bill Aulet, the Managing Director of the Marin Trust Center for the Massachusetts Institute of Technology, is as follows:

[C]reating value with new products, new ways of running businesses, and with a number of assets that [the company or organization] control. [19]

The concept of intraprenuership has always existed in AMCs as quality improvement and process improvement projects. The specialists who have led these projects in the past would be an ideal foundational group for a digital health development team in an AMC. However, as health care technology often integrates with existing EMR capabilities and may require bidirectional data flow from the record, it is critical to include a dedicated group of information technology (IT) specialists as collaborators. Additionally, this team will need training in implementation science, which focuses on converting research findings and evidence-based practices and implementing them into routine practice to impact patient care [20]. This training will enable the team to understand how to create digital health solutions that solve the problems faced by frontline health care providers or patients. Finally, these intraprenuership teams will require members with strong clinical experience or ad hoc subject matter experts to ensure the innovations being developed will either improve clinical care quality, efficiency in processes, or patient safety. Often the best innovators can create tools that fail to help the target audience after they implement phases; therefore, repeated experimentation in the form of pilot studies is critical to success [21]. Many AMCs have developed innovation centers to facilitate this work, but these centers will only be successful with adequate funding to enable development of the digital health solutions. If successful, these solutions can also outgrow into separate companies, be sold to other health care organizations, and serve as a form of revenue for the founding AMC.

If AMCs collaborate with entrepreneurs outside of the organization, such as health technology start-up companies, they can consider either codeveloping a digital health solution or adopting an existing solution [22]. As mentioned by Marwaha et al [23], some of the pitfalls when adopting an existing solution is the lack of understanding of the AMC’s problems and of the existing IT infrastructure in which the digital health tool must integrate. If codeveloping with a start-up company, the AMC may consider investing in the start-up, which will be high financial risk but can also result in a future source of revenue [23]. When codeveloping digital health solutions with a start-up company, AMCs have the added ability to provide clinical expertise as well as gain access to data and resources to help the start-up succeed while simultaneously customizing the solution to their own organization. The potential risk of adopting or partnering with digital health technology start-up companies is the Silicon Valley fail fast mentality [24]. Failing fast is ideal for early-stage start-up companies and venture capital firms; however, this often leaves early adopters without functioning technology and wasting their own resources on an innovation that either no longer exists or is no longer supported. If switching to an early-stage digital health solution, AMCs may disrupt existing workflows or technologies they use to provide patient care with the hope of improved efficiency or outcomes. Furthermore, there is significant up-front cost in the form of financial resources and time to integrate a new digital health tool into the current IT systems. Any collaboration, co-development, or investment by an AMC in an external company for digital health innovation must be vetted carefully. This ensures the digital health solution will resolve issues faced by the AMC and the external company will be dedicated to continual support and ongoing development of the innovation. With health care organizations in the United States already spending approximately 2.5%-2.8% of their annual revenue on IT costs, it is critical to ensure any added cost will be worthwhile for the long term [25].

Whether AMCs choose to develop in-house digital health innovations or integrate technology from external companies, they will need the full support of the organization’s senior leadership team. Many AMCs have incorporated innovation into their values and strategic foci, such as Cedars-Sinai and Brigham and Women’s Hospital, which enables conversations with board members and financial officers to provide funding to support these endeavors [26,27]. Also, it is critical to vet any new partnerships, ideas, and spun-off companies through the AMC’s legal department and ensure any research studies are approved through the organization’s IRB for clinical studies.

Despite AMCs comprising only 6% of the United States’ health care system, they have large unique patient data sets, advanced health information management systems, data abstraction infrastructure, a constant desire to improve patient care quality, research missions, and the scientific method mindset [6]. These characteristics make AMCs a unique and ideal environment for intrapreneurship or collaboration with external companies to develop digital health solutions that can be validated to ensure they improve efficiency, patient safety, and clinical care quality for patients. As the “third wave” of digital health innovation begins to swell, AMCs should lead this transformation for all health care.

## Abbreviations

AMC: academic medical center
EMR: electronic medical record
IRB: institutional review board
IT: information technology
